# Dual-Targeting Polymer Nanoparticles Efficiently Deliver DNA Vaccine and Induce Robust Prophylactic Immunity against Spring Viremia of Carp Virus Infection

**DOI:** 10.1128/spectrum.03085-22

**Published:** 2022-09-08

**Authors:** Chen Zhang, Peng-Qi Zhang, Sheng Guo, Zhao Zhao, Gao-Xue Wang, Bin Zhu

**Affiliations:** a College of Animal Science and Technology, Northwest A&F University, Yangling, Shaanxi, People’s Republic of China; Nanjing Institute of Geography and Limnology, Chinese Academy of Sciences

**Keywords:** spring viremia of carp virus, SVCV, DNA vaccine, polymer nanovaccine, antigen presenting, targeted delivery, anti-SVCV immunity

## Abstract

Spring viremia of carp virus (SVCV) is highly contagious and lethal to most cyprinid fish, causing serious economic losses to the carp aquaculture industry. Although DNA vaccines can generate long-term humoral and cellular immune responses, which provide protective immunity against SVCV, the major drawback of DNA vaccines is their low immunogenicity in clinical tests. Here, we construct a dual-targeted polymer DNA vaccine delivery platform (MCS-PCHG) by using mannosylated chitosan to encapsulate the poly(d,l-lactide-*co*-glycolide)-loaded DNA vaccine containing the heavy-chain C_H_3 region (CH3) of common carp IgM and the antigenic domain (G131c). The developed nanovaccine delivery platform showed good biocompatibility *in vivo* and *in vitro*. With the modification of the mannose moiety and the modification of CH3, the constructed MCS-PCHG could efficiently activate the maturation of antigen-presenting cells. Moreover, we observe significantly high level of immune-related genes expression, serum antigen-specific IgM, SVCV-neutralizing antibody titers in fish vaccinated with MCS-PCHG. Next, the protective efficacy of MCS-PCHG was further evaluated by challenge test. The highest survival rate (ca. 84%) was observed in fish vaccinated with MCS-PCHG after challenging with SVCV. This study presents a novel design for smart, dual-targeted polymer nanoparticles, which are inherently biocompatible, promising for targeted vaccine delivery.

**IMPORTANCE** Spring viremia of carp virus (SVCV) affects global cyprinid fish farming industry, with no available commercial vaccine. Herein, we developed a dual-targeting polymer nanovaccine (MCS-PCHG) by using mannose and common carp IgM heavy chain C_H_3 region (CH3) as antigen presenting cell (APCs) recognition moiety, attaining the effective delivery of antigen. This dual-targeting polymer vaccine can efficiently activate the APCs, and further induce robust and durable adaptive immune response with good protection against SVCV infection. Our study provides valuable theoretical basis for developing efficient vaccine against infectious diseases in aquaculture.

## INTRODUCTION

Spring viremia of carp (SVC) caused by a viral pathogen spring viremia of carp virus (SVCV) have emerged as a serious threat to the cyprinid fish cultivation industry worldwide, causing substantial economic losses (1, 2). It has been proved that DNA vaccine is one of the most promising applications in combating infectious pathogens including SVC and other viral diseases, due to its remarkable ability to generate long-term humoral as well as cell-mediated immune responses (3–5). However, its effectiveness in clinical trial is rather disappointing. DNA vaccine still remains some drawbacks, including the easily degradation by DNases and lysosomes, poorly distribution and inefficiently expression after injected with DNA vaccines and so on, which hamper its clinical development (6, 7). To promote efficient delivery of DNA vaccines into desired cells and protect them from degradation, efficient DNA vaccine delivery system is urgently needed.

Many polymers have been utilized to design nanoparticles (NPs) for DNA vaccine delivery, including poly(d,l-lactide-*co*-glycolide) (PLGA) and chitosan (CS) (8–10). PLGA is a kind of biodegradable, biocompatible, and nontoxic polymer that consists of three different hydroxyl acid monomers (d-lactic, l-lactic, and glycolic acids) (11). PLGA has been considered as an attractive carrier and adjuvant for vaccine antigens (12). PLGA NPs could prolong the antigen circulating time, as well as increase antigen stability, leading to increased immunity (13). Chitosan is a product of natural polysaccharide chitin with some acetyl groups removed, which has various well-known properties such as biodegradability, biocompatibility, nontoxicity, bioadhesion, and immune enhancement (14). Due to these characteristics, chitosan has been regarded as a promising drug/antigen carrier for delivering proteins, peptides, nucleic acids, and so on (15, 16). In addition, as a natural positively charged polymer with unique polymeric cationic characteristic gel-forming and film-forming properties, chitosan has been extensively utilized as a suitable candidate for gene delivery system (17).

Antigen-presenting cells (APCs) play a role in the vaccine-induced adaptive immunity (18). There are various important receptors on the surfaces of APCs, including mannose receptors, Fc receptors, Toll-like receptors, scavenger receptors, asialoglycoprotein receptors and so on (19). Among these receptors, mannose receptor is a vital pattern recognition receptor and endocytic receptor with multiple extracellular structural domains that recognizes and binds a wide range of endogenous and exogenous ligands (20). In addition, due to its high-affinity and high-level expression on the surfaces of APCs, mannose receptor is widely used as the target for antigen delivery (21). Fc receptors are receptors for the C terminus of the Fc portion of immunoglobulin (22). After the immunoglobulin (Ig) binds to the antigen, the Fc segment of the antibody transforms and binds to the Fc receptor on the cell membrane, producing various biological effects (23). The effects of the antigen-antibody complex are mediated after the combination between the C_H_3 domain of the immunoglobulin and Fc receptor on the APCs, so the Fc receptor plays an important role in inducing and regulating the immune response (24).

In this study, we formulated a dual-targeting polymer nanovaccine (MCS-PCHG) by using mannosylated chitosan-coated PLGA nanoparticles loaded with DNA vaccine encoding the antigen (G131c) of a highly contagious and lethal disease (Spring viremia of carp, SVC), and a heavy chain C_H_3 region (CH3) of carp immunoglobulin M (IgM). The dual-targeting polymer nanovaccine is consisted with two targeted ligands, the mannose could specifically bind with the mannose receptor on the surface of APCs; after translation and expression in the host, the CH3 could interact with the Fc receptor on the APCs. To evaluate the feasibility of targeted delivery of the constructed dual-targeting polymer nanovaccine, an environmental and economically important disease (SVC) was used as a model. Herein, we provide a novel design of smart dual-targeted polymer nanoparticles (MCS-PCHG) with are inherently biocompatibility, which could promote the activation of APCs and induce robust adaptive immune response against SVCV infection.

## RESULTS

### Synthesis and characterization of nanovaccines.

**FIG 1:**
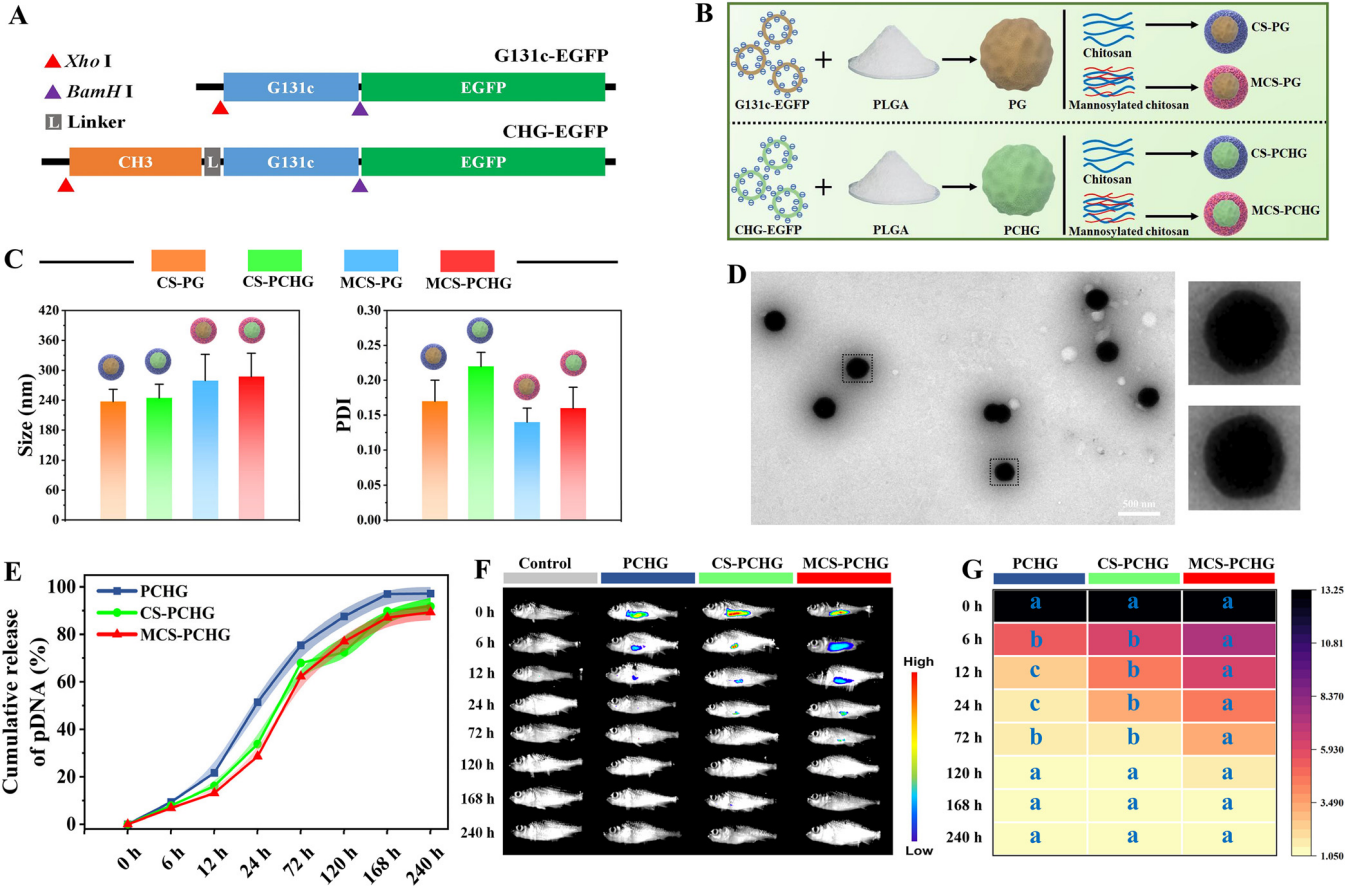
Molecular design and characterization of dual-targeting polymer nanovaccine. (A) Schematic diagram of pEGFP-G131c and pEGFP-CHG recombinant plasmids. (B) Schematic illustration of mannosylated chitosan/chitosan-based nanovaccine. (C) Hydrodynamic sizes of CS-PG, CS-PCHG, MCS-PG, and MCS-PCHG samples. Data are represented as means ± the SD (*n* = 3). (D) Morphology observation by TEM of MCS-PCHG. (E) pDHA release of PCHG, CS-PCHG, and MCS-PCHG nanovaccine with incubation at 37°C for 240 h. Data are represented as means ± the SD (*n* = 3). (F) Biodistribution of RBI-PCHG, RBI-CS-PCHG, and RBI-PCHG nanovaccine in vaccinated fish at different time points. (G) Quantitative fluorescence signals in different vaccinated fish. Data are represented as means ± the SD (*n* = 3). Different lowercase letters (a, b, and c) indicate significant differences (*P < *0.05).

The construction of the recombinant plasmids is described in Fig. 1A. The construction strategy of the polymer NPs is illustrated in Fig. 1B; in this design, G131c or CHG plasmids were loaded by PLGA NPs and then encapsulated with chitosan or mannosylated chitosan, respectively. The DNA loading capacity and encapsulation efficiency in the constructed PG-DNA NPs were determined to be ca. 0.5% and ca. 63.9%, respectively. The constructed CS-PG, CS-PCHG, MCS-PG, and MCS-PCHG show mean sizes of 237.42 ± 24.36 nm, 244.62 ± 27.69 nm, 279.36 ± 52.72 nm, and 287.62 ± 46.56 nm, respectively (Fig. 1C). Meanwhile, all of the constructed polymer NPs have a very compact size distribution with polydispersity indices (PDIs) ranging from 0.15 to 0.23 (Fig. 1C). As revealed by transmission electron microscopy (TEM), MCS-PCHG has a spheroidal NP structure (Fig. 1D). We further analyzed the pDNA release of the NPs *in vitro*. In the early stages (0 to 6 h) and the late stage (168 to 240 h), PCHG, CS-PCHG, and MCS-PCHG displayed similar pDNA release rates. From 6 h to 168 h, all of these NPs showed sustained rapid pDNA release; moreover, PCHG (without mannosylated chitosan/chitosan coating) exhibited a higher release rate than did CS-PCHG and MCS-PCHG (Fig. 1E). The delivery regulations of polymer NPs were determined by *in vivo* fluorescence imaging. As depicted in Fig. 1F and G, at the initial vaccination (0 h) and at 120 h postvaccination, fish vaccinated with rhodamine B isothiocyanate (mock), CS-PCHG, and MCS-PCHG showed similar fluorescence intensities. From 6 to 72 h postvaccination, fish vaccinated with MCS-PCHG showed higher vaccine retention contents than for the CS-PCHG- and mock-treated groups (*P < *0.05).

### Effects of MCS-PCHG on DNA vaccine delivery.

**FIG 2:**
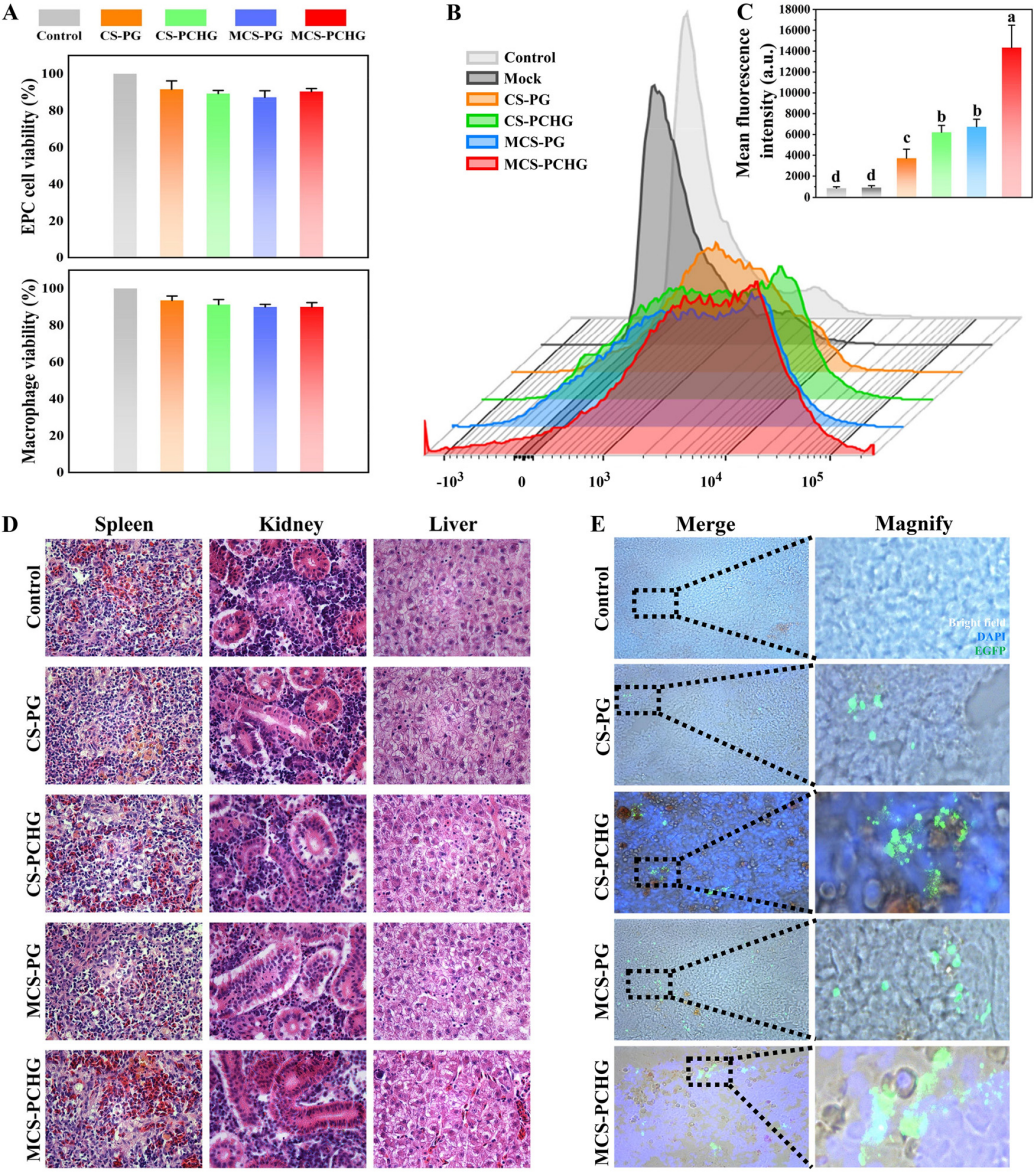
*In vitro* and *in vivo* uptake of the constructed polymer nanovaccines. (A) Cell viability of different nanovaccines. (B) Flow cytometry analysis of cellular uptake of nanovaccine by macrophages. (C) MFI values according to flow cytometry results. Statistical analysis was determined using Tukey’s test (bars represent means ± the SD) (*n* = 3). Different lowercase letters (a, b, and c) indicate significant differences (*P < *0.05). (D) Histological examination by H&E staining of spleens, kidneys, and livers from control and vaccinated fish (*n* = 3). (E) Detection of antigen proteins in carp spleen on day 7 postimmunization.

We further analyzed the effects of MCS-PCHG on DNA vaccine delivery *in vitro* and *in vivo*. First, the cytotoxicity of constructed nanovaccines was evaluated by incubation with EPC cells and macrophages for 24 h, respectively (Fig. 2A). An MTT [3-(4,5-dimethyl-2-thiazolyl)-2,5-diphenyl-2*H*-tetrazolium bromide] assay demonstrated no measurable impact of the nanovaccines on cell viability. Next, the cellular uptake of the MCS-PCHG was evaluated by using a flow cytometer. As shown in Fig. 2B and C, compared to the control group, the treatment groups (CS-PG, CS-PCHG, MCS-PG, and MCS-PCHG) showed significantly enhanced cellular uptake. Specifically, the mean fluorescence intensity (MFI) between CS-PG and CS-PCHG showed no significant differences; meanwhile, a similar result was observed in the MFIs between MCS-PG and MCS-PCHG. It is worth noting that the MFI of mannosylated chitosan-coated nanoparticles (MCS-PG and MCS-PCHG) was significantly higher than that for chitosan-coated nanoparticles (CS-PG and CS-PCHG).

Prior to the evaluation on the expression of antigen proteins in vaccinated fish, we analyzed the histological changes in fish spleens, kidneys, and livers after vaccination. As shown in Fig. 2D, no obvious pathological change was observed in these tissues after vaccination. Afterward, we examined the expression of antigen protein in vaccinated fish spleen with a confocal laser microscope. Since the expressed recombinant antigen protein contains green fluorescent protein, the intensity of the fluorescence reflects the content of the antigen protein to some extent. As depicted in Fig. 2E, stronger green fluorescence was observed in MCS-PCHG-treated carp spleen compared to other NP-treated groups, demonstrating the efficient delivery ability of MCS-PCHG *in vivo*.

### MCS-PCHG nanovaccine upregulates the expression levels of immune-related genes.

**FIG 3:**
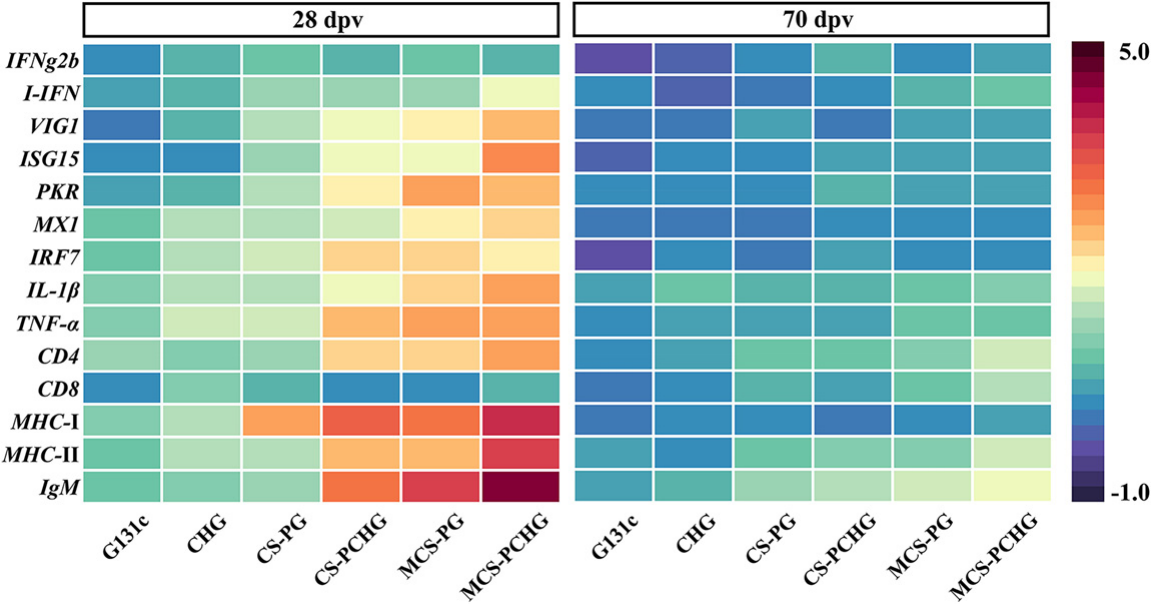
Immune gene expression in carp spleen. A heat map illustrates the expression profiles of immune genes in carp spleens at 28 and 70 dpv (*n* = 5). Color values correspond to the log_2_ fold change.

To evaluate the kinetics of the immune response in vaccinated carp, we used qPCR to analyze the expression of immune genes including interferon (*IFNg2b* and *I-IFN*), interferon regulatory factors (*IRF7*), virus inhibitory protein genes (*VIG1*, *ISG15*, and *PKR*), myxcovirus resistance 1 (*MX1*), cytokines (*TNF-α* and *IL-1β*), antigen-presenting-related genes (*CD4*, *CD8*, *MHC*-I, and *MHC*-II), and immunoglobulin heavy-chain gene (*IgM*) in spleen tissues at 28 days postvaccination (dpv) and 70 dpv (Fig. 3). Compared to the control group, our results showed that the levels of most of the genes discussed above involved in innate and adaptive immunity increased significantly after vaccination. Interestingly, the expression of these genes in the same treatment group at different time points was different; the fold changes for these genes at 28 dpv were much higher than at 70 dpv. Importantly, the greatest expression of these genes was observed in the MCS-PCHG group. Together, these data indicated that MCS-PCHG could efficiently induce innate and adaptive immune responses in the systemic immunity of carp.

### The MCS-PCHG nanovaccine potently activate APCs and induce robust adaptive immunity against SVCV infection.

**FIG 4:**
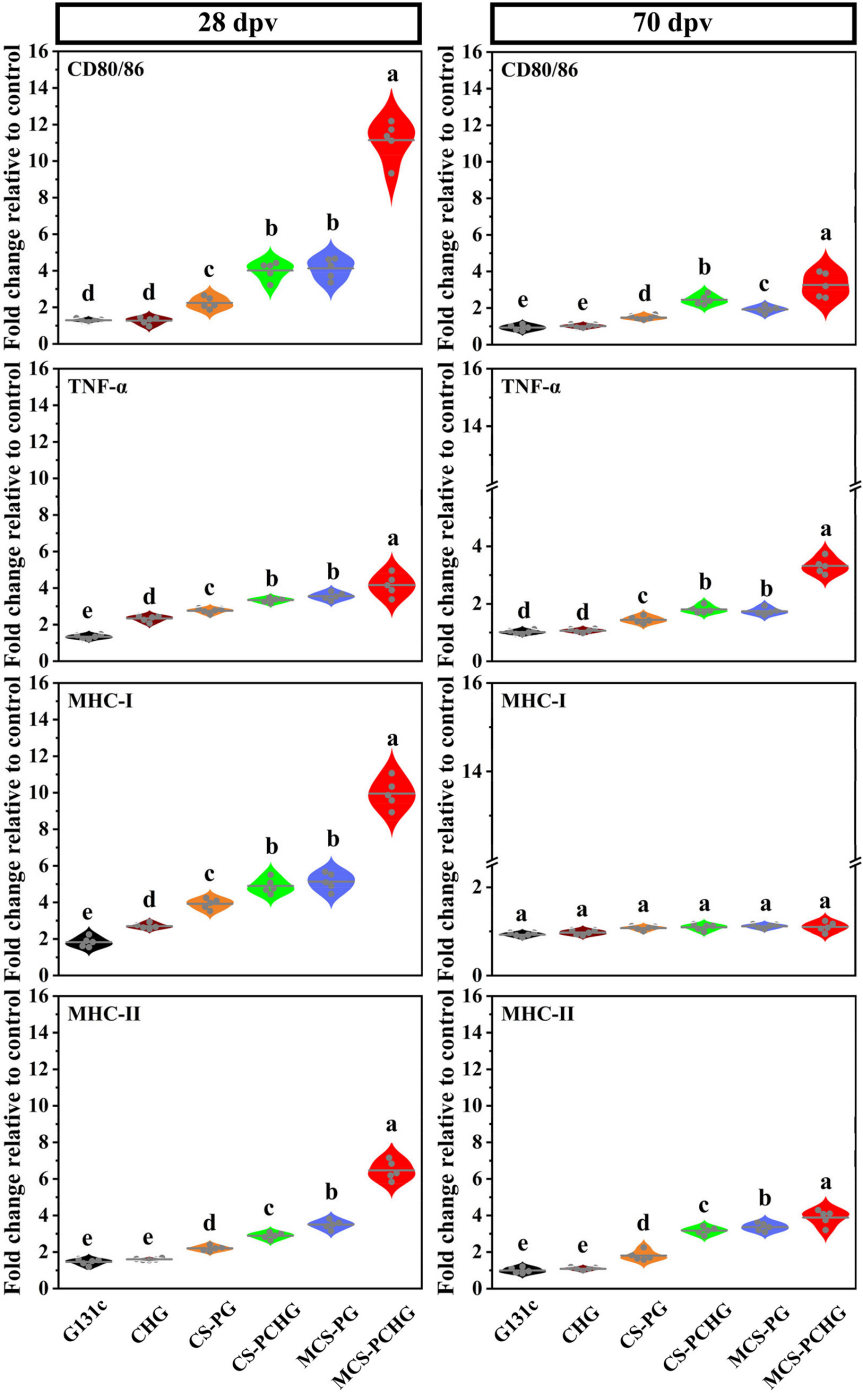
Relative expression of CD80/86, TNF-α, MHC-I, and MHC-II in vaccinated fish spleens at 28 and 70 dpv shown as violin graphs. The means are represented by central solid lines on each plot (*n* = 5). Different lowercase letters (a, b, c, d, and e) denote statistically significant differences between different groups (*P < *0.05).

It is important for immune responses that antigens are processed and presented by APCs. First, the abilities of NPs to stimulate APC maturation were evaluated by enzyme-linked immunosorbent assay (ELISA). We analyzed the secretion of CD80/86, tumor necrosis factor alpha (TNF-α), major histocompatibility complex class I (MHC-I), and MHC-II (Fig. 4). Compared to samples treated with other NP formulations, including CS-PG, CS-PCHG, and MCS-PG, MCS-PCHG showed significantly increased secretion levels of CD80/86, TNF-α, MHC-I, and MHC-II at 28 dpv. We obtained similar results in vaccinated fish at 70 dpv, significant increase of CD80/86, TNF-α, and MHC-II were observed in MCS-PCHG group.

**FIG 5:**
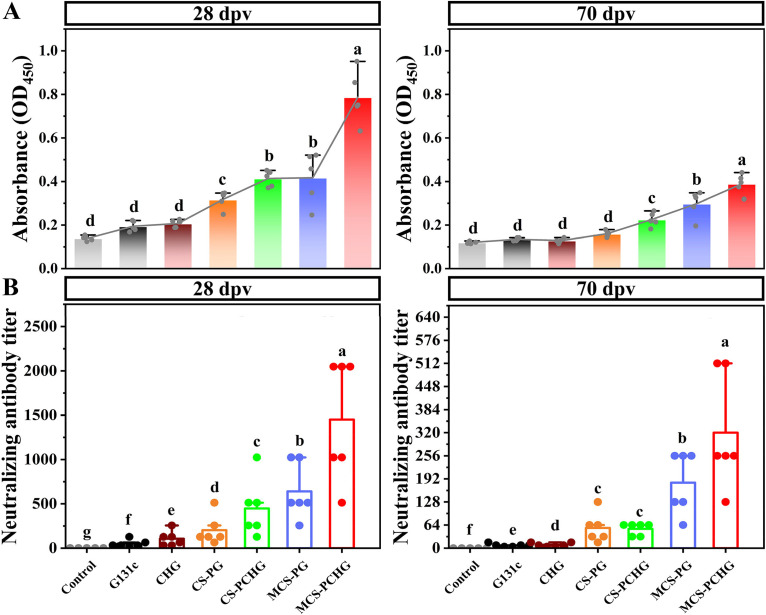
Antigen-specific antibody levels and serum neutralizing antibody levels in vaccinated carp. (A) ELISA was performed to determine the expression levels of antigen-specific antibody in carp serum (*n* = 5) at 28 and 70dpv, respectively. (B) A neutralizing antibody assay was performed to determine the inhibitory ability of each group of serum (*n* = 6) against SVCV on days 28 and 70, respectively. The data are expressed as means ± the SD. Different lowercase letters (a, b, c, d, e, f and g) indicate significant differences (*P < *0.05).

We further analyzed the antigen-specific IgM levels and neutralizing ability in vaccinated fish serum (Fig. 5). The results showed that significant levels (*P < *0.05) of antigen-specific IgM antibody were induced in MCS-PCHG vaccinated fish at 28 and 70 dpv compared to other NPs, including CS-PG, CS-PCHG, and MCS-PG. We further evaluated the neutralizing ability of serum antibodies isolated from each group. MCS-PCHG showed the highest neutralizing antibody titer, which was significantly higher than for any other treatment group (*P < *0.05).

**FIG 6:**
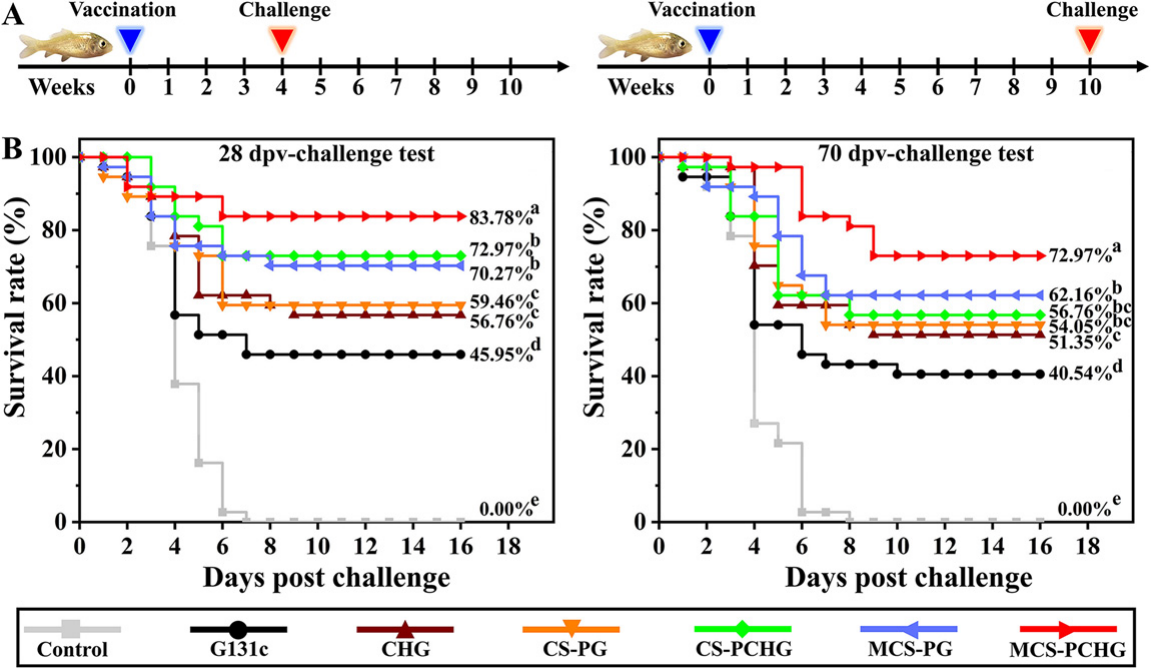
Evaluation on the protective effect of constructed polymer nanovaccines. (A) Schematic diagram of challenge strategy. (B) Survival rate of vaccinated carp after SVCV challenge. Different lowercase letters (a, b, c, and d) denote statistically significant differences between different groups (*P < *0.05).

Next, we evaluated the immunoprotection of the constructed nanovaccine against SVCV infection. As illustrated in Fig. 6, we performed two virus challenge tests at different time points. One challenge was performed on day 28. The highest survival rate (ca. 84%) was observed in MCS-PCHG group; it was significantly greater (*P* < 0.01) than that of fish in the control group. Moreover, at 70 dpv, the MCS-PCHG group showed significantly improved survival (relative to the control group), with the highest survival rate of ~73%. Altogether, these results suggest that a MCS-PCHG nanovaccine could efficiently activate APCs and induce robust prophylactic immunity against SVCV infection.

## DISCUSSION

Although DNA vaccination is considered as the potently strategy against various diseases, including spring viremia of carp virus, rabies virus, human immunodeficiency virus, hepatitis B, cancers, and so on, its clinical applications are confined by the low immunogenicity due to the rapid degradation and systemic clearance (25, 26). To address the limitations of DNA vaccine in clinical trial, herein, we developed a dual-targeting polymer nanoparticles by using PLGA and chitosan as the DNA vaccine carrier with the modification of two target ligands (mannose and the heavy chain C_H_3 region of IgM). The constructed polymer nanoparticles showed longer antigen-release duration than naked DNA vaccine (Fig. 1). Importantly, all components within the constructed polymer vaccine are biodegradable and have a good biosafety. Polymers, especially PLGA and chitosan, have been widely used as DNA vaccine carriers. PLGA is a kind of polymeric organic compound with excellent biocompatibility and degradability (27, 28). As a DNA vaccine vector, PLGA has the advantages including slow release, targeting, protection of DNA plasmid from destruction, high encapsulation carrying capacity, and easy escape from lysosomes (29). To prolong the retention of nanovaccine in the host, chitosan was wrapped around the surface of PLGA particles. As a desirable candidate for nucleic acids delivery, chitosan with the cationic nature has many promising properties such as nontoxicity, low-immunogenicity, and mucosal-adhesion (30). The positively charged property of chitosan could promote antigen presentation, moreover, the bioadhesive property enables the prolonged retention of chitosan-based nucleic acids delivery system (31, 32).

The process of antigen being efficiently endocytosed by the APCs largely determining the efficacy of vaccine (33). To facilitate the recognition and uptake by APCs, we modified the DNA loaded chitosan-PLGA particle with two target ligands (mannose and heavy chain C_H_3 region of IgM). There are many specific recognition receptors distributed on the surface of APCs, including mannose receptor, Fc receptor, toll-like receptor and so on (34). Mannose receptor can recognize a variety of molecules on the cell surface or the cell wall of pathogens, and maintain the balance of the internal environment by participating in receptor-mediated endocytosis and phagocytosis (35). Moreover, it bridges innate immunity with acquired immunity (36). Mannose receptor is widely used as the target for targeted vaccine delivery (37, 38). To further enhance the antigen presenting process, we added another target ligand (heavy chain C_H_3 region of IgM). The heavy chain C_H_3 region of IgM could specifically recognized by the Fc receptor on the surface of APCs (39). As expected, the dual-targeting polymer nanovaccine showed enhanced APCs uptake and the following superior APCs-maturation induction capacity (Fig. 2 and 4). Therefore, the mannosylated modification and the addition of the heavy chain C_H_3 region of IgM could be an effective strategy for vaccine targeted delivery to APCs.

We further evaluated the immune responses induced in vaccinated fish. The expression level of immune-related genes reflects the level of vaccine-induced immune response to a certain extent. We detected the relative expression levels of immune-related genes of *IFNg2b, I-IFN, VIG1, ISG15, PKR, MX1, IRF7*, *IL-1β, TNF-α, CD4, CD8,* *MHC*-I, *MHC*-II, and *IgM* in the spleen of vaccinated fish by qRT-PCR at 28 and 70 dpv, respectively. Compared with the control groups, the expression levels of all genes were significantly up-regulated in the MCS-PCHG immunized groups at 28 dpv. Similar results were found in MCS-PCHG group at 70 dpv. Interferons (*IFNg2b* and *I-IFN*) and interferon stimulated genes (including *VIG1, ISG15, PKR,* and *IRF7*) play critical role in antiviral immunity in vertebrates (40, 41). After virus infection, vaccination or treatment with poly I:C, the expression of these genes could be significantly increased (42, 43). TNF-α and *IL-1β* are pro-inflammatory cytokines, which act as modulators of the host innate and adaptive immune responses (44). CD4, CD8, MHC-I, and MHC-II are important molecules involving antigen presenting process (45). Teleost IgM are important components of adaptive immunity (46). We demonstrated that the expression of antigen-specific IgM was significantly upregulated in fish serum and spleen after fish vaccinated with MCS-PCHG, moreover, the SVCV neutralizing antibody titer was significantly increased in MCS-PCHG group. We further evaluated the protective effects of the constructed nanovaccine. After challenge, the protective effect of MCS-PCHG is stronger than that of other treatments, achieving the highest survival rate of ca. 84%. All these results greatly support the feasibility and validity of our design on nanovaccine involving the PLGA and chitosan as the vaccine carrier, mannose and carp IgM heavy chain C_H_3 region as the APC recognition moiety to further promote vaccination efficacy.

## MATERIALS AND METHODS

### Materials.

PLGA was purchased from Top Science Biological Product Co., Ltd. (Beijing, China). Chitosan was purchased from Shandong AK Biotech, Co., Ltd. (Shandong, China). Mannosylated chitosan was purchased from SunLipo NanoTech Co., Ltd. (Shanghai, China). Rhodamine B isothiocyanate (CAS number 36877-69-7) was purchased from Sinopharm Chemical Regent Co., Ltd. (Shanghai, China). PicoGreen (CAS number 177571-06-1) was purchased from Henan Bon Industrial Co., Ltd. (Henan, China).

### Experimental animals.

SVCV-free common carp (~1.2 g and ~300 g) were obtained from a commercial fish company (Guangzhou, China) and reared in a recirculated system (Shanghai Haisheng, China) at 25 to 27°C and a pH of 7.0 to 7.4, with dissolved oxygen at >6.0 mg/mL and nitrogen at <0.05 mg/mL. Fish were fed with commercial pelleted dry feed (ZaiChong, China).

All animal experiments were conducted in accordance with the *Guide for the Care and Use of Northwest A&F University*. The animal use protocol was reviewed and approved by the Animal Ethical and Welfare Committee, Northwest A&F University, China (approval DK2021001).

### Cells, virus, and plasmids.

Carp macrophages were isolated from the head kidneys of common carp (~300 g) according to a previous method (47). EPC cells were cultured in medium 199 containing glucose and l-glutamine (M199; HyClone, USA) and supplemented with 10% fetal bovine serum (FBS; Every Green, China), penicillin (100 IU/mL), and streptomycin (0.1 mg/mL) at 25°C in 5% CO_2_ incubator. SVCV (conserved in our laboratory) was used for viral infection. Viral titers were determined by the Reed and Muench method (48). Recombinant plasmid pEGFP-G131c containing a truncated SVCV *glycoprotein* gene (*G131c*) and recombinant plasmid pEGFP-CHG containing *G131c* and the heavy-chain C_H_3 region (accession number AB004108.1) of common carp IgM were constructed by Genecreate Co., Ltd. (Wuhan, China).

### Preparation and characterization of the nanoparticles.

By using a double-emulsion method (49), plasmids pEGFP-G131c and pEGFP-CHG were loaded using PLGA to form PG and PCHG nanoparticles, respectively. The chitosan/mannosylated chitosan coated nanoparticles were constructed as previously reported (50). Briefly, the prepared PG and PCHG compound emulsions were added to chitosan or mannosylated chitosan acetic acid solution containing 0.5% polyvinyl alcohol, respectively. Then, we volatilized the organic solvent at room temperature and washed the samples with double-distilled water, followed by centrifugation at 12,000 rpm three times. The obtained polymers were dissolved in deionized water and centrifuged at 4,500 rpm for 20 min to discard the large particles.

The concentration of the nanoparticle pDNA was analyzed by using the PicoGreen method (51), and results were calculated using a standard curve (*R*^2^ = 0.9963) as follows: pDNA concentration (ng/mL) = (fluorescence value – 146.15)/42.65. Rhodamine B isothiocyanate-labeled nanovaccines (RBI-PCHG, RBI-CS-PCHG, and RBI-MCS-PCHG) were constructed as described previously (49).

A light-scattering particle sizer (Zetasizer Nano ZS90TM; Malvern, England) was used to analyze the particle sizes and the polydispersity indices (PDIs) of the prepared nanoparticles. A TEM microscope (TF20; FEI, Netherlands) was used to analyze the morphology of the constructed nanovaccines.

### *In vitro* and pDNA release study.

An *in vitro* release study was performed as previously reported (49). Briefly, prepared nanovaccine dry powder (containing 100 μg of pDNA) was added to 1 mL of phosphate-buffered saline (PBS; pH 7.4) with continuous shaking at 100 rpm/min at 37°C. The solution was then centrifuged at different time points (0, 6, 12, 24, 72, 120, 168, and 240 h) at 12,000 rpm/min, respectively. The pDNA in the supernatant was measured by using the PicoGreen method (51).

### *In vivo* delivery of constructed nanoparticles.

Common carp (~1.2 g) were randomly selected and intraperitoneally injected with RBI-labeled nanovaccines (RBI-PCHG, RBI-CS-PCHG, and RBI-MCS-PCHG) at doses of 2 μg/g (body weight), respectively. The living body imaging system AniView 100 (BLT, China) was used to detect RBI-labeled nanovaccines at different time points (0, 6, 12, 24, 72, 120, 168, and 240 h) postvaccination. To quantify the content of nanovaccines in different tissues, AniView 100 Living Imaging software was used.

### Biosafety analysis.

The safety of the constructed nanovaccines was evaluated *in vivo* and *in vitro*. To evaluate the constructed nanovaccine *in vivo*, healthy common carp (*n* = 50 per group) were intraperitoneally injected with CS-PG, CS-PCHG, MCS-PG, and MCS-PCHG at a concentration of 2 μg of pDNA/g of fish, respectively. The health status of the treated fish was recorded for a period of 2 months postvaccination. The spleens, kidneys, and livers in treated fish at 70 dpv were sampled and histologically analyzed using hematoxylin and eosin (H&E) staining, as previously reported (47).

To determine the cytotoxicity of the nanovaccines (CS-PG, CS-PCHG, MCS-PG and MCS-PCHG), carp macrophages and EPC cells were seeded in 96-well plates at a density of 1 × 10^5^ cells per well, followed by incubation overnight. Different nanovaccines at concentrations of 20 μg/mL were incubated with these cells for 24 h. The relative cell viabilities were determined by an MTT assay (Sigma, USA) according to the standard protocol.

### The cellular uptake of the constructed nanovaccine by carp macrophages.

Macrophages were seeded in 12-well plates and grown to a monolayer. The macrophages were incubated with the RBI-labeled nanovaccines (RBI-CS-PG, RBI-CS-PCHG, RBI-MCS-PG, and RBI-MCS-PCHG) at a concentration of 10 μg/mL for 6 h, respectively. Untreated macrophages served as a control. The fluorescence intensity of internalization from RBI-labeled nanovaccines in macrophages was measured by using FACSAria (BD, USA) flow cytometry.

### Vaccination and challenge.

SVCV-free common carp (~1.2 g) were used for vaccination. Fish were randomly divided into seven groups (100 fish per group): PBS, G131c (pEGFP-G131c plasmid), CHG (pEGFP-CHG plasmid), CS-PG, CS-PCHG, MCS-PG, and MCS-PCHG. All fish were vaccinated by intraperitoneal injection at a dose of 2 μg/g (body weight). Subsequently, the fish were transferred to different tanks and monitored daily.

For the challenge test, the water temperature was kept at 17 ± 0.5°C. Two independent challenge tests were carried out 28 and 70 days dpv, respectively. Each group of 50 vaccinated fish was randomly selected, transferred to new tanks, and intraperitoneally injected with 20 μL of 10^2^ × 50% tissue culture infective dose(s) (TCID_50_)/mL of SVCV. The survival rate was calculated after 16 days postchallenge.

### RNA isolation and quantitative real-time PCR analysis.

Spleens were sampled at 28 and 70 dpv from vaccinated common carp, respectively. For RNA isolation and cDNA synthesis cDNA synthesis, total RNAs were obtained from the spleen in each group (*n* = 3 per group) with TRIzol reagent. HiScript Q Select RT SuperMix for aPCR (+gDNA wiper; Vazyme, China) was performed to reverse transcribe the purified RNA into cDNA. qRT-PCR was performed in triplicate using the CFX96 real-time PCR detection system (Bio-Rad, USA) with AceQ qPCR SYBR Green Master Mix (Vazyme, China). The qRT-PCR procedure consisted of an initial denaturation step at 95°C for 5 min, followed by 40 cycles of 95°C for 15 s and then 57°C annealing for 1 min. All qRT-PCRs were performed for three biological replicates and repeated with two independent samples. The *β-actin* gene was used as an internal control (Table 1). The relative mRNA expression was calculated using the 2^–ΔΔ^*^CT^* method (52) according to the following formula: F = 2^–ΔΔ^*^CT^*, where ΔΔ*C_T_* = (*C_T_*_, target gene_ – *C_T_*_, reference gene_) – (*C_T_*_, target gene_ – *C_T_*_, reference gene_) control.

**TABLE 1:** Primers used for the analysis of mRNA expression by qRT-PCR

Gene	Accession no.	Primer	
		Orientation	Sequence (5′–3′)
*SVCV N*	NC002803.1	Forward	AACAGCGCGTCTTACATGC
		Reverse	CTAAGGCGTAAGCCATCAGC
*β-actin*	M24113	Forward	GCTATGTGGCTCTTGACTTCG
		Reverse	CCGTCAGGCAGCTCATAGCT
*40S*	AB012087.1	Forward	CTCTGCCAAATCACCATACTC
		Reverse	GCGGTTTTCTGTATGTGTCTC
*IFNg2b*	JX181980.1	Forward	GCTCAAGAAGTATGCAGAAACTC
		Reverse	TCTGGCTTGTCGTCTCCT
*I-IFN*	AB376666.1	Forward	CAGAGTCAATGCTCCGCTT
		Reverse	CTCAGATGACTGCCGTTGC
*VIG1*	JX131617.1	Forward	CGCACCAAAGAGCAGAAAGA
		Reverse	AATGGGCAAGACGAAAGAGG
*ISG 15*	KP115358.1	Forward	AAGCCATATTCAGCGAAGC
		Reverse	AACCGTTATCGGCAGACAG
*PKR*	EX880666.1	Forward	CCAACATCGTCCGCTACTACTC
		Reverse	GCGTGTCTCCCTCACAAAG
*MX1*	KP115357.1	Forward	GGCTGGAGCAGGTGTTGGTATC
		Reverse	TCCACCAGGTCCGGCTTTGTTAA
*IRF 7*	JQ698666.1	Forward	TCCACTGAGGGTCTGATTGA
		Reverse	CGCTGGTGCTGACGAAGA
*IL-1β*	AJ245635	Forward	GACTTTTTAATGTTTGTGGG
		Reverse	CATAAATGTAAATGTCAACACCCTTC
*TNF α*	JN412133.1	Forward	CTGGTGATGGTGTCGAGGAGGA
		Reverse	CTGAGACTTGTTGAGCGTGAA
*CD4*	DQ400124	Forward	AAGGTGCCGGCTGTGAT
		Reverse	TAAGGTTTTTGTGTTATATTGTTTTG
*CD8*	XM042759578	Forward	TGGCTTTTCTTCCCAAAGC
		Reverse	AACAGGGTTAAAACAACTGGATTCC
*MHC-*I	XM_042729998.1	Forward	ATGATGATTCAAAACACTACGAC
		Reverse	TAATTCACATCCAGCAAGTCTCTGGTGAACAT
*MHC-*II	S62611.1	Forward	TGCAGTGCCTATGACTTC
		Reverse	GAGCTGGCGTGCTCCA
*IgM*	AB004105	Forward	CACAAGGCGGGAAATGAAGA
		Reverse	CTGATAAAGCTTTGCACTTCAGCA

### ELISA analysis.

On days 28 and 70 after postimmunization, serum was collected from common carp according to a previously reported method (49). The ELISAs for CD80/86, TNF-α, MHC-I, and MHC-II expression in treated fish were analyzed by using fish CD80/86, TNF-α, MHC-I, and MHC-II ELISA kits (Renjiebio, China), respectively. The expression of serum antigen-specific IgM was analyzed by ELISA according to a previous method (53). Briefly, the 96-well ELISA plate was coated with SVCV at 4°C for 12 h, followed by incubation with 5% skimmed milk in PBS (pH 7.4) for 2 h at 37°C. Each well was washed with PBST and incubated with serum samples (diluted at 1:50) as the primary antibody, followed by incubation at 37°C for 2 h and then washing with PBST. After that, horseradish peroxidase-conjugated anti-common carp (*Cyprinus carpio carpio*)/koi carp (*Cyprinus carpio koi*) IgM monoclonal antibody (Aquatic Diagnostics, Ltd., England) diluted at 1:1,000 was used as the secondary antibody. Tetramethylbenzidine (Tiangen, China) was used as a colorimetric substrate, and the color was developed. The absorbance at 450 nm was measured by using a microplate reader (Molecular Devices Corp., Palo Alto, CA).

### SVCV neutralization assay.

Sera from vaccinated and untreated carp at 28 and 70 dpv were used to analyze the neutralizing ability against SVCV, as previously described (54). Briefly, 50 μL of serum from each carp (*n* = 6 per group) was subjected to a 2-fold serial dilution in a 96-well plate and then mixed fully with the same volume of SVCV (100 TCID_50_/well) at 28°C for 2 h. The mixture was then added to another 96-well plate containing a confluent monolayer of EPC cells, followed by incubation at 28°C for 1.5 h. The infected EPC cells were next washed three times with sterile PBS and cultured with M199 medium containing 2% FBS for 6 days. The wells were scored for cytopathic effect, and a neutralization titer was calculated as the reciprocal of the highest serum dilution at which full virus neutralization occurred.

### Statistical analysis.

Data are presented as means ± the standard deviations (SD). SPSS 15.0 software (SPSS, Inc., USA) was used to perform statistical analysis. Statistical differences between different groups were analyzed by using one-way analysis of variance (ANOVA), followed by Tukey’s test. Different lowercase letters in the figures indicate significant differences (*P < *0.05).
